# Climate extremes are associated with invertebrate taxonomic and functional composition in mountain lakes

**DOI:** 10.1002/ece3.2517

**Published:** 2016-10-17

**Authors:** Kate S. Boersma, Avery Nickerson, Clinton D. Francis, Adam M. Siepielski

**Affiliations:** ^1^ Department of BiologyUniversity of San DiegoSan DiegoCAUSA; ^2^ Department of Biological SciencesCalifornia Polytechnic State UniversitySan Luis ObispoCAUSA; ^3^ Department of Biological SciencesUniversity of ArkansasFayettevilleARUSA

**Keywords:** aquatic ecology, aquatic invertebrate, biodiversity, climate variability, community structure, extreme events, functional diversity, global change, lentic, macrophyte

## Abstract

Climate change is expected to increase climate variability and the occurrence of extreme climatic events, with potentially devastating effects on aquatic ecosystems. However, little is known about the role of climate extremes in structuring aquatic communities or the interplay between climate and local abiotic and biotic factors. Here, we examine the relative influence of climate and local abiotic and biotic conditions on biodiversity and community structure in lake invertebrates. We sampled aquatic invertebrates and measured environmental variables in 19 lakes throughout California, USA, to test hypotheses of the relationship between climate, local biotic and environmental conditions, and the taxonomic and functional structure of aquatic invertebrate communities. We found that, while local biotic and abiotic factors such as habitat availability and conductivity were the most consistent predictors of alpha diversity, extreme climate conditions such as maximum summer temperature and dry‐season precipitation were most often associated with multivariate taxonomic and functional composition. Specifically, sites with high maximum temperatures and low dry‐season precipitation housed communities containing high abundances of large predatory taxa. Furthermore, both climate dissimilarity and abiotic dissimilarity determined taxonomic turnover among sites (beta diversity). These findings suggest that while local‐scale environmental variables may predict alpha diversity, climatic variability is important to consider when projecting broad‐scale aquatic community responses to the extreme temperature and precipitation events that are expected for much of the world during the next century.

## 1 Introduction

A growing body of research suggests that climate variability is increasing (Easterling et al., 2000; Huntington, 2006; Karl, Meehl, & Miller, 2008; Ruff, Kushnir, & Seager, 2011), producing more extreme precipitation and temperature events. These climate extremes may be equally important determinants of biological organization as changing mean environmental conditions (Boucek & Rehage, 2014; Lloret, Escudero, Iriondo, Martínez‐Vilalta, & Valladares, 2012). Recent case studies have shown that extreme climatic conditions frequently determine organismal fitness (Easterling et al., 2000; Kingsolver et al. 2012; Paaijmans et al. 2013; Vasseur et al. 2014), which should in turn determine local species persistence and thus community makeup. Despite this evidence, the implications of climate extremes for the structure of biological communities remain largely unknown (Diez et al., 2012; Lynch et al., 2013; Vázquez, Gianoli, Morris, & Bozinovic, 2015; Williams & Jackson, 2007). Specifically, we do not yet understand how climate extremes interact with local biotic and abiotic drivers of community structure (Jentsch, Kreyling, & Beierkuhnlein, 2007; Wernberg, Smale, & Thomsen, 2012) especially in freshwater systems.

Conversely, the role of the local biotic and abiotic environment in structuring aquatic communities is well documented. For example, interspecies interactions such as predation (Shurin et al., 2002; Terborgh, 2015) and herbivory (Hairston & Hairston, 1993) are strong determinants of community structure. Likewise, the local abiotic environment is known to affect aquatic communities through water temperature, conductivity, dissolved oxygen, and other physical and chemical factors (Dossena et al., 2012; Kratina, Greig, Thompson, Carvalho‐Pereira, & Shurin, 2012; Ledger, Brown, Edwards, Milner, & Woodward, 2013; Ledger, Edwards, Brown, Milner, & Woodward, 2011; Schindler et al., 1990; Woodward et al., 2012).

Although abiotic, biotic, and climate effects can be studied separately, it is more appropriate to study them together due to the simple reality that abiotic and biotic factors interact with one another over long‐term climate regimes (Dunson & Travis, 1991). The importance of this integrated approach becomes obvious when trying to understand how climate extremes may affect community structure. Changes in climate can affect biotic interactions that structure communities, such as competition and predation (Greig, Wissinger, & McIntosh, 2013; Tylianakis, Didham, Bascompte, & Wardle, 2008), and characteristics of the local environment may complicate seemingly straightforward associations between climate and local abiotic conditions (Fey, Mertens, Beversdorf, McMahon, & Cottingham, 2015a; Hwan & Carlson, 2016). Ecologists have increasingly adopted this integrative approach; however, much of our understanding of how communities are structured still focuses on the importance of average conditions and does not account for *variability* in climate or in the local environment (Thompson, Beardall, Beringer, Grace, & Sardina, 2013).

Climate and local environmental conditions can affect multiple dimensions of biodiversity (Leary, Rip, & Petchey, 2012). This includes major biogeographic patterns such as species richness (alpha diversity) and turnover in species composition among sites (beta diversity). They also impact variation in functional traits of species within communities and in trait composition across communities (Boucek & Rehage, 2014). Despite an increasing number of studies that integrate taxonomic and trait information, the relationship between the set of environmental factors that shape taxonomic diversity and those that determine functional diversity is still poorly understood. This is especially true in relation to variation in climate factors. Environmental characteristics can be expected to filter species from the regional species pool based on their traits, allowing only a subset to persist in a given location (Webb, Hoeting, Ames, Pyne, & Poff, 2010). Therefore, different factors acting on functional and taxonomic diversity could highlight community structuring mechanisms that act across taxonomic groups (Weiher & Keddy, 1995).

Manipulative experiments have been used to tease apart specific environmental drivers of community responses to climate perturbations (Fey & Cottingham, 2012; Greig et al., 2012; Jentsch et al., 2007), but mechanisms identified at small experimental scales may not fully explain broad landscape patterns in community structure (Kissling & Schleuning, 2014). While manipulative experiments are ultimately necessary to determine cause and effect, observational studies can lead to important insights because the effects of the biotic and abiotic environment are integrated over longer timescales and greater spatial extent than are often possible during experimental studies (Whittaker, Willis, & Field, 2001). Indeed, evaluating the role of extreme, rare events is often difficult because by definition they are rare (Fey et al., 2015b; Siepielski & Benkman, 2007). One approach to address this challenge is to pair observational studies with landscape‐scale climate records (e.g., Hijmans, Cameron, Parra, Jones, & Jarvis, 2005), which makes it possible to model the relationship between longer term climate variability that captures climate extremes and local environmental variables and community structure.

Aquatic invertebrate communities in mountain lakes of California, USA, are ideal for these investigations because (1) they encompass a wide range of spatial scales and climate patterns, (2) their communities are taxonomically diverse, and (3) associated climate records are readily available. We sampled aquatic invertebrates in these lakes and examined the local environment and climate as predictors of spatial patterns in taxonomic and trait diversity. In studies comprising multiple spatial scales such as ours, the scales of environmental predictors and community responses alone may account for observed patterns (Heino et al., 2015; Levin, 1992; Wiens, 1989). Therefore, we hypothesized that the scale of the diversity measurements would match the scale of the environmental predictors: (1) local biotic and abiotic factors will be the strongest determinants of local alpha diversity and community composition of taxonomy and traits, and (2) topographic and climate factors will be the strongest determinants of turnover among communities (beta diversity). We found that this was not entirely the case, and incongruences between our predictions and outcomes suggest an important role of climate extremes.

## 2 Materials and methods

### 2.1 Invertebrate sampling

**Figure 1:**
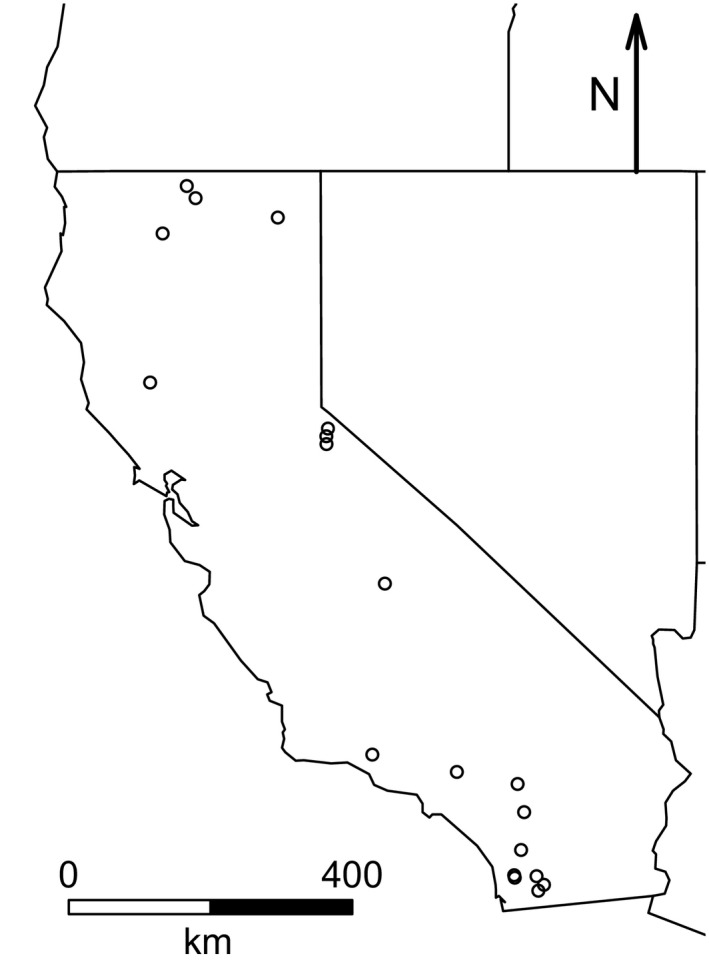
Locations of the lakes included in our study within the state of California, USA

We sampled aquatic invertebrates in 19 lakes throughout California, USA, during June and July of 2014 (Figure 1; Table 1). We selected lakes based on their accessibility, permanence, and the presence of macrophytes in the littoral zone. We targeted macrophytes for invertebrate sampling because emergent vegetation has been documented to be important habitat for a rich community of species (Beckett, Aartila, & Miller, 1992; Brown, Poe, French Iii, & Schloesser, 1988; Cyr & Downing, 1988; Gregg & Rose, 1985), and is the primary habitat for the most numerous invertebrate predatory taxa in our samples, damselflies in the family Coenagrionidae (Crowley & Johnson 1992). Invertebrates were collected using a 6‐L box sampler (100‐um mesh), which was placed over the macrophytes, allowing us to target taxa that live on and around the stems and leaves (Downing, 1986). Three box samples were taken per lake, and invertebrates were preserved in 70% ethanol. We subsampled each box to a threshold of 300 individuals and identified animals to the highest resolution possible given available resources (usually family, Merritt, Cummins, & Berg, 2008). Invertebrate abundances for the three box samples were averaged for each lake.

**Table 1:** Physical, geographic, and climate characteristics of the lakes included in this study

Lake	Latitude	Longitude	Elevation (m)	Lake area (m^2^)	Annual mean temperature (°C)	Annual precipitation (mm)
Blue Lake	41.415	−120.686	1,850	648,487	5.9	420
Blue Lake Road Pond	38.616	−119.916	2,452	2,998	4.2	1,035
Boulder Oaks	32.967	−116.929	450	3,997	16.6	430
Burnside Lake	38.714	−119.891	2,498	1,070	4	990
Camp Lake Sequoia	36.730	−118.988	1,630	322,424	10.1	824
Corte Madera Pond	32.799	−116.555	1,117	6,537	12.9	585
Crystal Lake	34.320	−117.847	1,688	34,029	10.4	826
Dos Picos	32.998	−116.938	454	8,851	16.5	439
Gumboot Lake	41.211	−122.512	1,861	43,612	5.2	954
Jenk's Lake	34.165	−116.884	2,051	37,741	8.5	656
Juanita Lake	41.818	−122.129	2,931	226,508	6.1	453
Lake Cuyamaca	32.985	−116.583	1,415	395,706	12	736
Lake Fulmore	33.805	−116.780	1,632	12,573	11.3	677
Letts Lake	39.303	−122.710	1,381	129,313	9.4	1,263
Lower Rose Valley Lake	34.542	−119.187	1,019	20,738	13.3	547
Mendenhall Ranch	33.321	−116.828	1,368	5,292	12.8	663
Mosquito Lake	38.516	−119.914	2,464	13,972	4.1	1,042
Orr Lake	41.663	−121.989	2,787	245,920	7	439
Water of the Woods	32.875	−116.466	1,640	2,046	11.5	642

### 2.2 Environmental predictors

We assembled a set of 61 potential explanatory variables to develop models to explain variation in community structure across our lake samples. These variables encompassed a wide range of spatial and temporal scales and included factors that we measured *in situ*, such as water chemistry and habitat structure, and factors that we assembled from other sources such as land cover classes and climate variables.

#### 2.2.1 Local water chemistry and environmental variables

We recorded water temperature, dissolved oxygen, conductivity, and pH of three replicate water samples taken from the littoral zone of each lake using handheld probes (YSI model 85, YSI Incorporated, Yellow Springs, OH, USA; Milwaukee model SM102, Milwaukee Instruments incorporated, Rock Mount, NC, USA). We measured chlorophyll‐a in the water column by filtering 1000 ml of lake water through a glass fiber filter (47 mm, Pall Corporation, Ann Arbor, MI, USA), extracting the pigment for 24 hr at 4°C with 95% ethanol, and quantifying fluorescence using a fluorometer (Turner Designs, Sunnyvale, CA, USA). Raw fluorescence was then converted to μg/L chlorophyll‐a. We quantified macrophyte density in three quadrats (0.5 × 0.5 m) distributed approximately evenly along 10‐m transects though the littoral zone where samples were collected. We sampled fish using a 4.5 × 1.5 m beach seine (5 mm mesh), towed for 10 m through the water adjacent to the macrophyte beds. Three replicate tows were taken per lake; fish presence/absence was recorded, and then fish were released. Means of continuous variables were used in all analyses.

#### 2.2.2 Climate variables

We obtained climate data from WorldClim (Hijmans et al., 2005; www.worldclim.org) on aspects of each lake's precipitation and temperature regimes, including measures of both mean and extreme conditions (Table S1). We used a dataset representing “current” 50‐year average climate conditions (1950–2000) in high‐resolution grids (30 arc‐seconds), providing a spatial resolution of approximately 1 km.

#### 2.2.3 Land cover and other variables

Categorizing sites by land cover is a useful way to summarize land use and broad‐scale landscape heterogeneity (Homer, Huang, Yang, Wylie, & Coan, 2004), especially in our case since our lakes were distributed over a wide spatial and anthropogenic land use gradient. Regional land cover data were obtained from the 2011 National Land Cover Database (Homer et al., 2015) and are described in Table S2. We characterized land cover surrounding each sampling location with a buffer radius of 500 m, sampling 0.785 km^2^ for each location. This resolution struck a balance between our need to characterize larger scale patterns of land cover and the need to differentiate land cover characteristics among relatively close sites (minimum distance of 3.58 km). We also obtained human population density for subcounty partitions from the 2010 census from Census.gov. All spatial data were obtained using the function extract() in the raster 2.2‐31 package (Hijmans & van Etten, 2012) in R (R Core Team 2014) .

### 2.3 Alpha diversity metrics

We calculated species richness, taxonomic diversity (Hill numbers; Hill, 1973; Jost, 2006), and functional diversity (FD(Q); Chiu & Chao, 2014) for each box sample and then averaged the three samples for each lake. We selected Hill numbers to measure taxonomic diversity because they represent the species equivalents of standard entropy‐based diversity measurements such as Shannon index and are easily interpretable (Jost, 2009).

We also examined how functional trait diversity varied among sites. Species traits (e.g., body size and trophic level) determine how species respond to the environment and affect ecosystem functioning (Naeem & Wright, 2003), and trait‐based approaches allow for comparisons among communities without shared species (McGill, Enquist, Weiher, & Westoby, 2006; Messier, McGill, & Lechowicz, 2010). We used published resources (Boersma, Bogan, Henrichs, & Lytle, 2014a; Schriever et al., 2015) to compile information for the taxa in our samples on seven functional traits: body size, functional feeding group, locomotion, dispersal capacity, respiration mode, voltinism, and diapause (Table S3). These traits were selected to represent fundamental biological processes that are important in the structure of aquatic communities. We calculated functional diversity (FD(Q); Chiu & Chao, 2014) to quantify the total functional distance among all species in each lake community. FD(Q) is similar to Rao's Q (Botta‐Dukát, 2005) in that it is an abundance‐weighted index of the trait dissimilarity of species in a community, except that it is constructed from functional Hill numbers and thus is more interpretable (Pavoine, Marcon, & Ricotta, 2016). We measured dissimilarity in trait composition using Sorensen distance (Sørensen, 1948) to account for the non‐normal distributions and large number of zeros that characterize community data (McCune & Grace, 2002).

Predators have a fundamental role structuring communities (Nystrom, Svensson, Lardner, Bronmark, & Graneli, 2001; Terborgh, 2015). To explore the role of macroinvertebrate predators in determining prey community diversity, we also analyzed a matrix of prey taxa only. This allowed us to use variables associated with predator abundance and diversity as predictors. To do so, we created a second sample‐by‐species matrix after removing all predatory taxa. We calculated species richness, taxonomic diversity, and functional diversity for each box sample for both the complete matrix and the prey matrix. Then, we averaged these abundance and diversity values across the three samples within each lake to create two lake‐by‐species matrices: the complete community matrix and the prey matrix. We used both prey and complete matrices for our analyses of alpha diversity and community composition, described below.

Diversity metrics were calculated using the vegan (Oksanen et al., 2012) and FD (Laliberté & Shipley, 2011) package in R version 3.1.1 (R Core Team 2014).

### 2.4 Linear models

We used model selection to test hypotheses of the drivers of alpha diversity (richness, taxonomic diversity, and functional diversity). One of the ongoing challenges with applying linear modeling approaches to understand community structure is how to handle a large number of predictors (Cade, 2015; Hooten & Hobbs, 2015; Warton et al., 2015), because models that include many predictors are likely to detect spurious associations (Burnham & Anderson, 2002). In order to avoid spurious model selection outcomes, we simplified the list of predictors into reduced models representing our hypotheses (Burnham & Anderson, 2002) and then tested these hypotheses using AICc. We created four models of the drivers of alpha diversity: local biotic, local abiotic, climate, and topographic processes (Table 2). The first three models were created based on our questions regarding the relative influence of local and climate factors. The topographic model included spatial and land use variables such as land cover, geographic coordinates, and elevation.

**Table 2:** Model selection output. The four hypothesized models (local abiotic, local biotic, topographic, and climate) were tested as predictors of the three diversity metrics (taxonomic diversity, species richness, and functional diversity) for both the complete community matrix and a matrix of prey taxa only

Community	Response variable	Hypothesis	Candidate model and parameter significance	AICc	Delta AICc	Adj R^2^	F	*df*	*p*‐value
Entire	Taxonomic diversity	Local abiotic	Taxonomic diversity ~ WaterTemp + DOppm + log(Cond) + pH	83.31	5.06	–	–	–	–
		**Local biotic**	Taxonomic diversity ~ log(MacDensity)** + **ActualFishPA** + **chlA**	**78.25**	**0**	**.365**	**4.449**	**3, 15**	**.01997**
		Topographic	Taxonomic diversity ~ LAT + log(PopDensity) + Water + EvergreenForest	92.17	13.92	–	–	–	–
		Climate	Taxonomic diversity ~ AnnMeanTemp + TempAnnRange + AnnPrecip+ PrecipCV	84.99	6.74	–	–	–	–
	Species richness	**Local abiotic**	**Species richness ~ WaterTemp** + **DOppm** + log(Cond)** + **pH**	**95.66**	**0**	**.4039**	**4.049**	**4, 14**	**.02189**
		**Local biotic**	Species richness ~ log(MacDensity)** + **ActualFishPA** + **chlA**	**95.79**	**0.13**	**.2944**	**3.504**	**3, 15**	**.0418**
		Topographic	Species richness ~ LAT + log(PopDensity) + Water + EvergreenForest	103.86	8.2	–	–	–	–
		Climate	Species richness ~ AnnMeanTemp + TempAnnRange + AnnPrecip + PrecipCV	98.35	2.69	.3131	3.051	4, 14	.05292
	Functional diversity	**Local abiotic**	**Functional diversity ~ WaterTemp** + **DOppm** + log(Cond)** + **pH**	**188.58**	**0**	**.4271**	**4.355**	**4, 14**	**.01701**
		Local biotic	Functional diversity ~ log(MacDensity)* + ActualFishPA + chlA	190.84	2.26	.2415	2.91	3, 15	.06893
		Topographic	Functional diversity ~ LAT + log(PopDensity) + Water + EvergreenForest	197.55	8.97	–	–	–	–
		Climate	Functional diversity ~ AnnMeanTemp + TempAnnRange + AnnPrecip+ PrecipCV	194.43	5.85	–	–	–	–
Prey only	Taxonomic diversity	Local abiotic	Prey taxonomic diversity ~ WaterTemp + DOppm + log(Cond) + pH	80.4	7.77	–	–	–	–
		**Local biotic**	Prey taxonomic diversity ~ log(MacDensity)* + **log(MesopredSum** + **1)**+ **ActualFishPA** + **chlA**	**72.62**	**0**	**.4999**	**5.498**	**4, 14**	**.007111**
		Topographic	Prey taxonomic diversity ~ LAT + log(PopDensity) + Water + EvergreenForest	87.99	15.36	–	–	–	–
		Climate	Prey taxonomic diversity ~ AnnMeanTemp + TempAnnRange + AnnPrecip + PrecipCV	80.96	8.34	–	–	–	–
	Species richness	Local abiotic	Prey species richness~WaterTemp + DOppm + log(Cond) + pH	93.53	5.43	–	–	–	–
		**Local biotic**	Prey species richness~log(MacDensity)* + **log(MesopredSum** + **1).** + **ActualFishPA.** + **chlA**	**88.1**	**0**	**.5162**	**5.802**	**4, 14**	**.005729**
		Topographic	Prey species richness ~ LAT + log(PopDensity) + Water + EvergreenForest	99.32	11.21	–	–	–	–
		Climate	Prey species richness~ AnnMeanTemp + TempAnnRange + AnnPrecip+ PrecipCV	95.67	7.56	–	–	–	–
	Functional diversity	**Local abiotic**	**Prey functional diversity ~ WaterTemp** + **DOppm** + log(Cond)** + **pH**	**177.75**	**0.02**	**.4395**	**4.529**	**4, 14**	**.0148**
		**Local biotic**	**Prey functional diversity ~ log(MacDensity).** + **log(MesopredSum** + **1)** + **ActualFishPA.** + **chlA**	**177.73**	**0**	**.4402**	**4.539**	**4, 14**	**.1469**
		Topographic	Prey functional diversity ~ LAT + log(PopDensity) + Water + EvergreenForest	185.42	7.69	–	–	–	–
		Climate	Prey functional diversity ~ AnnMeanTemp + TempAnnRange + AnnPrecip + PrecipCV	184.19	6.46	–	–	–	–

Bold text indicates models with Delta AICc < 2.

*p* < .01 = **, *p* < .05 = *, *p* < .1 =.

We determined the subset of available variables to include in each of the four models using a combination of approaches. First, we examined collinearity among variables using scatterplots, correlation matrices, and variance inflation factors. For highly correlated variables, we selected representatives that explained the variability in the response, characterized biological processes of interest associated with freshwater community structure, and were easily interpretable. Correlations between the selected variables in the four models can be found in Table S4.

#### 2.4.1 Climate

Many of the climate variables were correlated (e.g., maximum temperature of the warmest month, maximum temperature of the warmest quarter, mean temperature of the driest quarter, annual mean temperature), so we removed variables with high variance inflation factors until we settled on a model that minimized collinearity and contained measures of both means and variability for the 50‐year time span of our climate data. This resulted in a candidate model that contained annual mean temperature, temperature annual range, annual precipitation, and the coefficient of variation of precipitation.

#### 2.4.2 Topographic

We selected two land cover variables to include in the topographic model: the proportion of evergreen vegetative cover and the proportion of land covered in water. Canopy cover is important for shading macrophyte beds (Köhler, Hachoł, & Hilt, 2010; Twilley & Barko, 1990) and contributing leaf litter to the lake detritus (Webster & Benfield, 1986), which can have strong effects on the composition of aquatic communities (Anderson & Sedell, 1979; Kelly, Bothwell, & Schindler, 2003). Evergreen trees were the dominant canopy in our study. We included water coverage to account for the overall amount of aquatic habitat available and also the degree of isolation and fragmentation of lakes, which affect the availability of sources for potential colonists and can influence community assembly dynamics (Chase, Bergett, & Biro, 2010; Michels et al., 2001). Due to the northeast‐to‐southwest arrangement of California's mountain ranges (Figure 1), latitude and longitude of our lakes were also highly correlated. We elected to include latitude because latitudinal diversity gradients are of interest in the general ecological literature (Gaston, 2000; Hawkins et al., 2003; Whittaker et al., 2001).

#### 2.4.3 Local biotic and local abiotic

The two local models contained all of the biotic and abiotic variables that we measured during sampling: water temperature, dissolved oxygen, conductivity, and pH in the local abiotic model, and the presence or absence of fish, chlorophyll‐a, and the density of macrophytes in the biotic model.

#### 2.4.4 Model selection

Following appropriate transformations (Table 2), we found that distributions of all variables approximated normality and subsequently used multiple linear regressions for the model selection procedure. Species richness is classically modeled with a Poisson distribution; however, in our case, we averaged the three box samples per lake prior to modeling so richness also approximated normality. We assessed the relative support for each of the candidate models using Akaike's information criterion with a correction for small sample sizes (AIC_c_) and the resulting ∆AIC_c_ values and Akaike weights (Burnham & Anderson, 2002). We considered models with ∆AIC_c_ of <2 to be equivalent. Model assessment was conducted using the leaps (Lumley, 2009) and AICcmodavg (Mazerolle, 2015) packages in R Version 3.1.1.

### 2.5 Community composition

Patterns of species co‐occurrence and functional diversity cannot be understood with summary metrics of alpha or beta diversity alone. Thus, we examined the relationship between the entire set of local, topographic, and climate predictors and community/trait composition with multivariate ordination methods. To prepare the lake‐by‐species matrix for ordination, we removed rare taxa (<3 individuals in all lakes) and singleton taxa (species found only in a single lake). The lake‐by‐species matrix was multiplied by the species‐by‐trait matrix to generate a lake‐by‐trait matrix that represented the relative abundance of each trait state in each lake (McCune & Grace, 2002). We applied a Wisconsin transformation to the species and trait matrices before ordinating (Legendre & Gallagher, 2001). We used pairwise Bray–Curtis distances between lakes for both species and trait analyses.

We ordinated the species and trait matrices using nonmetric multidimensional scaling (NMDS). This approach allowed us to examine associations between the entire suite of potential predictor variables and taxonomic and functional composition without first defining explicit hypotheses or removing correlated predictors. We examined stress values and convergence to assess ordination fit; two‐dimensional ordinations were appropriate in all cases. To facilitate comparisons of species and trait ordinations, we rotated each NMDS ordination to align with a vector representing the abundance of coenagrionid damselflies. Damselflies were the most numerous predatory taxon by a factor of 10, and previous studies have documented their fundamental role in aquatic food webs (Merritt et al., 2008). We examined species and trait correlations with the ordination space (considered meaningful when r* *>* *.5). We plotted significant associations between the potential predictors and the NMDS axes as vectors on the ordinations (*p *<* *.05, except where noted). We ordinated the full species/trait matrices and species/trait matrices without predatory taxa (four ordinations total) to allow us to examine the effect of predators as drivers of prey community and trait composition. We conducted all multivariate analyses in R package vegan (Oksanen et al., 2012) in R Version 3.1.1.

### 2.6 Beta diversity

Finally, we examined dissimilarity among sites in taxonomic and trait composition with beta diversity. Beta diversity is defined in many ways in the literature (Anderson et al. 2011). We were interested in beta diversity as turnover, which is the change in community composition among sample units along a gradient. Our aim was to determine which environmental gradient(s) best predicted turnover in taxonomic and functional diversity. To do so, we first generated Euclidean distance matrices representing the intersite dissimilarity in four predictor matrices of interest: local biotic, local abiotic, topographic, and climate. We added an additional distance matrix representing the Euclidean distance among sites (km), as geographic distance decay is often important for determining community differentiation and we needed to control for it in our models (Nekola & White, 1999; Soininen, McDonald, & Hillebrand, 2007). We corrected for the curvature of the earth using the Haversine formula (Shumaker & Sinnott, 1984). These five predictor matrices were assessed as predictors of two response distance matrices: taxonomic beta diversity and functional beta diversity. To calculate taxonomic dissimilarity for prey and complete matrices, we used Raup–Crick dissimilarity (Raup & Crick, 1979). This null‐model approach accounts for differences in alpha diversity among sites that may otherwise exaggerate beta diversity patterns (Chase, Kraft, Smith, Vellend, & Inouye, 2011). We calculated trait dissimilarity as the Euclidean distance between pairs of lakes based on the lake‐by‐trait matrix described under “Community composition.”

Next, we used partial Mantel tests to assess the capacity of the predictor distance matrices to predict beta diversity after controlling for geographic distance (Goslee & Urban, 2007). For taxonomic and functional beta diversity, we compared four candidate models, constructed from each of our four predictor matrices: community dissimilarity ~ predictor matrix + geographic distance. We assessed significance at an alpha of 0.05. Partial Mantel tests were conducted using the R package vegan (Oksanen et al., 2012).

## 3 Results

### 3.1 Alpha diversity

We identified 37 taxa in the 19 lakes. The most abundant taxon was Chironomidae, with an average of 232 individuals per box sample. Microcrustaceans (copepods, daphniids, and ostracods) were also numerous (mean abundances >50), as were physid and planorbid snails (mean abundances >30). The number of individuals per sample varied from 59 to 6,728 (mean = 812), and six taxa appeared in only a single sample (Corydalidae, Tipulidae, Dixidae, Crambidae, Sphaeriidae, and Viviparidae). The wide range of abundances was mirrored by high interlake variability in all three diversity metrics: species richness (mean = 9.51, *SD* = 3.25), taxonomic diversity (mean = 4.57, *SD* = 1.82), and functional diversity (mean = 58.21, *SD* = 32.30).

Model selection largely supported our hypothesis that biotic and abiotic factors would determine taxonomic and functional alpha diversity (Table 2). The local biotic model was preferred over local abiotic, topographic, and climate models for taxonomic diversity, and biotic and abiotic models were nearly equivalent at predicting functional diversity and species richness. These patterns held for both analyses including the entire community and the prey community alone (Table 2).

When we examined individual environmental variables within each preferred model, macrophyte density was a significant predictor of taxonomic diversity and richness, and conductivity predicted richness and functional diversity. In the prey models, mesopredator abundance was a significant predictor of taxonomic diversity. Complete model selection results are presented in Table 2.

### 3.2 Community composition

**Figure 2:**
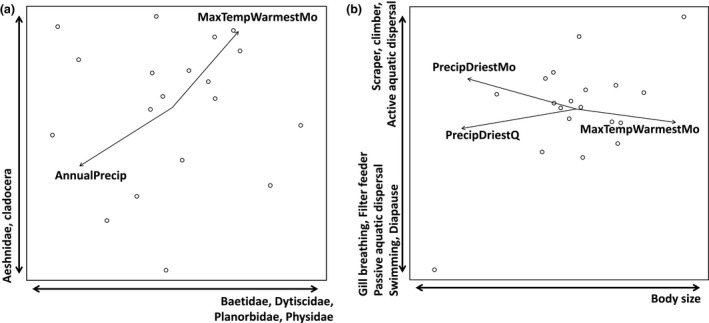
Nonmetric multidimensional scaling ordination of lakes by their taxonomic and trait composition. (a) Taxonomic ordination (NMDS: k = 2, stress = 0.1872), (b) trait ordination (NMDS: k = 2, stress = 0.1264588). Vectors represent significant correlations between biotic, abiotic, topographic, or climate variables and the ordination space (Pearson correlation: *p* < .05). When vectors overlapped on the ordinations, the vector with the lowest *p*‐value is displayed. Influential species/traits are indicated along each axis (|r| > .5). Each ordination was rotated so that its first axis is parallel to a vector of damselfly abundance (“Coenagrionidae”) to facilitate comparison between plots

Variables representing climate extremes were important for both taxonomic and trait composition. Specifically, of all of the potential local, topographic, and climate variables, maximum temperature of the warmest month and mean annual precipitation were found to be significantly correlated with the taxonomic NMDS ordination space (Table 3; Figure 2a). Taxa that were positively associated with Axis 1 included predatory diving beetles (Dytiscidae), mayfly prey (Baetidae), and the planorbid and physid snails (Pearson r: .5854, .5130, .5832, and .5802, respectively). Predatory dragonflies (Aeshnidae) and daphniid plankton prey (Cladocera) were negatively associated with Axis 2 (r: −.6591, −.5419).

**Table 3:** Vector correlations with NMDS ordination axes (Figure 2 and Fig. S1). Vectors are listed for the entire community ordination with correlations at *p* < .05 and for the prey ordination at *p* < .01. When vectors overlapped, the vector with the lowest *p*‐value is displayed on the ordinations and indicated here by bold text. Abbreviations are described in Tables S1 and S2

Community	Ordination	Correlated variable	R^2^	*p*‐value
Entire	Taxonomy	**MaxTempWarmestMo**	**.5859**	**.006**
		**AnnPrecip**	**.4958**	**.002**
		PrecipColdestQ	.4927	.003
		PrecipWettestMo	.4781	.005
		PrecipWettestQ	.4745	.006
	Traits	**PrecipDriestQ**	**.4153**	**.014**
		**PrecipDriestMo**	**.391**	**.014**
		**MaxTempWarmestMo**	**.3083**	**.045**
Prey	Taxonomy	**PrecipDriestQ**	**.6286**	**.001**
		**AnnPrecip**	**.5727**	**.002**
		**PrecipWettestMo**	**.5627**	**.001**
		PrecipColdestQ	.5511	.001
		PrecipWettestQ	.5299	.003
		PrecipWarmestQ	.4618	.007
		**PrecipDriestMo**	**.4569**	**.008**
		**MaxTempWarmestMo**	**.4572**	**.008**
	Traits	**DamselSum**	**.546**	**.011**
		**EvergreenForest**	**.495**	**.003**
		**ShrubScrub**	**.4503**	**.006**
		**PrecipDriestQ**	**.3943**	**.016**
		**PrecipDriestMo**	**.3689**	**.021**

Similar to the taxonomic ordination, climate extremes were also associated with the trait ordination. Dry‐season precipitation and the maximum temperature of the warmest month were significantly correlated with trait composition (Table 3). Body size was an influential trait on Axis 1 of the trait ordination, and large body sizes were associated with high temperatures and low precipitation (Figure 2b; r: .6320). Influential traits for Axis 2 included many traits that are associated with adaptation to life in an aquatic environment, including swimming capacity (r: −.680), passive and active aquatic dispersal (r: −.569 and .561), and filter feeding (r: −.721).

Taxonomic and trait ordinations of the prey community largely highlighted the same patterns as those of the entire community (Table 3 and Fig. S1). Overall, the ordinations revealed consistent associations between community composition and precipitation and temperature extremes.

### 3.3 Beta diversity

After controlling for geographic distance, we found that among‐site dissimilarity in climate was a significant predictor of taxonomic dissimilarity (Mantel test: r* *=* *.3277, *p *=* *.001), as was local abiotic dissimilarity (r* *=* *.3183, *p *=* *.03); however, biotic dissimilarity and topographic dissimilarity were not (biotic: r* *=* *.06264, *p *=* *.334, topographic: r* *=* *.07431, *p *=* *.317). Thus, spatial variation in taxonomic beta diversity was largely determined by climate and local abiotic factors.

The functional beta diversity analysis was less conclusive. After controlling for the effects of distance, there were no significant associations between functional diversity and any of the four predictor matrices. Euclidean distance was the only significant predictor of functional dissimilarity between sites, albeit weakly (r* *=* *.16709853, *p *=* *.034).

## 4 Discussion

We detected a consistent relationship between climate and the taxonomic and trait structure of aquatic invertebrate communities. Specifically, measures of the variability of temperature and precipitation—not mean values—were associated with multivariate trait composition among communities. Additionally, dissimilarities in climate and local abiotic conditions were better predictors of community turnover among sites than the dissimilarity of either the biotic environment or topographic characteristics. Despite a signal of climate extremes in multivariate community structure and beta diversity, biotic and abiotic local environmental conditions were the best predictors of alpha diversity at the local scale. In conjunction with previous studies, we found that the taxonomic alpha diversity of aquatic invertebrate communities is strongly shaped by the biotic environment (Shurin, Gruner, & Hillebrand, 2006).

### 4.1 Climate variability

Our study provides evidence that climate extremes are associated with functional trait composition (Figure 2b). Specifically, precipitation during the driest quarter and maximum temperature during the warmest month were significantly correlated with the trait ordination space, suggesting that these variables may be important determinants of spatial variation in trait composition. Logic dictates that communities in naturally variable regions are more likely to contain taxa that are adapted to fluctuating environmental conditions. As a result, these communities may have high resistance and resilience to future extreme events (Boersma et al., 2014a; Bogan, Boersma, & Lytle, 2015), and there is growing evidence that community responses to extreme climate events are governed by the functional diversity of local communities (Boucek & Rehage, 2014; Kreyling, Jentsch, & Beierkuhnlein, 2011). One prediction of climate change models is an increase in the frequency, duration, and magnitude of extreme climatic events (Easterling et al., 2000; IPCC 2012; Karl, Knight, & Plummer, 1995; Seager et al., 2007, Sydeman, Santora, Thompson, Marinovic, & Lorenzo, 2013; Vano et al., 2014), and recent research suggests that increasing variability in environmental conditions can be as important in determining organismal responses as changing mean values (Kayler et al., 2015; Thompson et al., 2013), especially in aquatic systems (Calapez, Elias, Almeida, & Feio, 2014; Chessman, 2015; Szczerkowska‐Majchrzak, Lik, & Leszczyńska, 2014). Climate extremes like high temperature and low precipitation, such as we identified in this study, likely limit the diversity of aquatic species at a site to a subset of the regional species pool that can survive local conditions (Bogan et al., 2015; Boulton & Lake, 2008; García‐Roger et al., 2013). An understanding of this climate‐driven filtering process is necessary to predict how ecosystems may respond to future climate disturbances (Webb et al., 2010). Our study advances this understanding by identifying extreme dry‐season precipitation and temperature as potentially important variables in this filtering process.

Maximum temperature of the warmest month was also positively correlated with the abundance of large predatory taxa (Figure 2b and Fig. S1a). There is evidence that warming can strengthen trophic cascades in pond food webs by increasing the effects of fish on primary productivity (Kratina et al., 2012), and top predators may mediate the effects of climate on the rest of the biotic community (Boersma, Bogan, Henrichs, & Lytle, 2014b). Our results suggest that future increasing maximum summer temperatures may increase the abundance of predatory taxa. Changing distribution and abundance of dominant predators may further amplify the strength of top‐down effects (Baum & Worm, 2009; Otto, Berlow, Rank, Smiley, & Brose, 2008). Ultimately, this could fundamentally restructure food webs.

Despite the consistency of the climate signal in our analyses of beta diversity and community composition, climate did not predict local alpha diversity. Our climate data were compiled from 50 years of observations taken at a relatively coarse spatial resolution given the size of our lakes (Hijmans et al., 2005) and therefore may lack site‐specific information necessary to predict alpha diversity. Additionally, our family‐level taxonomic resolution may have obscured a signal of climate in local communities. It is also possible that the effects of climate on local diversity are propagated through changes to local biotic and abiotic factors as has been observed in other studies (Boersma et al., 2014b; Kratina et al., 2012). Future investigations that use local weather station records and species‐level taxonomic information may be necessary to understand the role of climate at the local scale.

### 4.2 Local biotic factors

We confirm the conclusions of previous studies that local biotic factors, such as predation and productivity, are important drivers of aquatic biodiversity (e.g., Chase, 2007; Hairston & Hairston, 1993; Terborgh, 2015). Our local biotic model consistently outperformed topographic and climate models, with biological variables explaining between 30% and 52% of the variation in alpha diversity. Of the variables in the local biotic model, macrophyte density was the most consistent predictor. Macrophytes are home to complex food webs (Newman, 1991) and provide important structure for both grazing herbivores and sit‐and‐wait predators (Gregg & Rose, 1985; Warfe & Barmuta, 2004). The density of macrophytes varied widely among our lake samples, and our results reflect the influence of physical structure on invertebrate communities.

As expected, predator abundance predicted prey taxonomic diversity in the linear models (Table 2). In the multivariate analyses of prey community composition, large predatory dragonflies (Aeshnidae) and diving beetles (Dytiscidae) had the strongest associations with the ordination axes of any other taxon (Figure 2a), representing their influence on community structure. Aeshnid dragonflies are opportunistic predators of coenagrionid damselflies and other mobile taxa (McPeek, 1990), and dytiscid beetles are generalist predators and scavengers (Hicks, 1994; Obha, 2009; Velasco & Millan, 1998). These findings support recent assertions of the strength of top‐down processes at determining community structure (Boersma et al., 2014b; Terborgh, 2015). Further research is needed to determine whether biotic and climate drivers interact synergistically to amplify their effects on functional diversity and trait composition.

### 4.3 Beta diversity

Taxonomic dissimilarity among lakes was best predicted by intersite dissimilarity in climate and local abiotic variables, whereas functional dissimilarity was predicted by Euclidean distance alone. Among‐site differences in species composition can be determined by many concurrent forces (Cottenie, 2005; Heino et al., 2015; Leibold & McPeek, 2006; Siepielski & McPeek, 2013). Our results suggest that species sorting along environmental gradients may play an important role in determining the structure of lake invertebrate fauna. We suspect that climate extremes act to filter the overall regional species pool and yield a subset of regional diversity that can survive in a given area. Then, local biotic interactions among this subset of species are most important at the local level. However, the lack of an association between functional trait dissimilarity and climate, abiotic, biotic, or topographic dissimilarity suggests that this “filtering” process is not based on the traits included in this analysis.

Our study contributes to the growing number of empirical investigations that document the potential role of climate extremes in shaping community structure (Jentsch et al., 2007; Thompson et al., 2013; Vázquez et al., 2015) and the association between climate and functional traits (Barrows, Rotenberry, & Allen, 2010; Brown & Milner, 2012; Buisson, Grenouillet, Villeger, Canal, & Laffaille, 2013). While much needs to be done to understand the fundamental role of climate variation in structuring communities, recent theoretical work suggests that increasing environmental variability and novel climate disturbance regimes can surpass species’ abilities to respond via phenotypic plasticity or adaptive evolution. Botero et al. (2015) show that even very minor changes in environmental conditions can constitute “tipping points” between plasticity and population collapse. These tipping points also act at the community level and can fundamentally restructure food webs (van Nes & Scheffer, 2004). Indeed, our results suggest that temperature and precipitation extremes may have important roles in structuring communities. Moreover, embedded within this larger scale climate variation, local abiotic and biotic factors further act on constituent species to govern the makeup and spatial variation of the communities we documented. Understanding this complex interplay between climate extremes at the regional level and abiotic and biotic factors at the local level is necessary for a more complete view of how and why communities are structured as they are and how they may change in response to novel future conditions.

## Conflict of Interest

None declared.
