# Supplementary material for: Climate extremes are associated with invertebrate taxonomic and functional composition in mountain lakes
Source: Ecol Evol. 2016 Oct 17;6(22):8094–106. doi: 10.1002/ece3.2517 (PMC5108261; doi:10.1002/ece3.2517)
Supplement: Supplementary file 1 [file ECE3-6-8094-s001.docx]

**Supporting information for Boersma et al.**

Table S1. Climate variables. Information obtained from WorldClim ([Hijmans et al. 2005; www.worldclim.org](#_ENREF_39)) representing 50-yr average climate conditions (1950-2000) for each of the study locations.

| Abbreviation | Climate variable |
| --- | --- |
| AnnMeanTemp | Annual mean temperature |
| MeanDiurnalTempRange | Mean diurnal temperature range |
| Isothermality | Mean diurnal temperature range / Temperature annual range |
| TempSD | Temperature standard deviation |
| MaxTempWarmestMo | Maximum temperature of the warmest month |
| MinTempColdestMo | Minimum temperature of the coldest month |
| TempAnnRange | Temperature annual range (max temp - min temp) |
| MeanTempWettestQ | Mean temperature of the wettest quarter |
| MeanTempDriestQ | Mean temperature of the driest quarter |
| MeanTempWarmestQ | Mean temperature of the warmest quarter |
| MeanTempColdestQ | Mean temperature of the coldest quarter |
| AnnPrecip | Annual precipitation |
| PrecipWettestMo | Precipitation of the wettest month |
| PrecipDriestMo | Precipitation of the driest month |
| PrecipCV | Precipitation coefficient of variation |
| PrecipWettestQ | Precipitation of the wettest quarter |
| PrecipDriestQ | Precipitation of the driest quarter |
| PrecipWarmestQ | Precipitation of the warmest quarter |
| PrecipColdestQ | Precipitation of the coldest quarter |

Table S2. Land cover variables obtained from the 2011 National Land Cover Database ([Homer et al. 2015](#_ENREF_41)). We characterized regional land cover surrounding each sampling location with a buffer radius of 500 m.

| Land cover variable | Description |
| --- | --- |
| Water | Open water with less than 25% cover of vegetation or soil. |
| Developed Open Space | Mostly lawn grasses, impervious surfaces <20% of total cover. |
| Developed Low Intensity | A mixture of constructed materials and vegetation, impervious surfaces 20-49% of total cover. |
| Developed Medium Intensity | A mixture of constructed materials and vegetation, impervious surfaces 50-79% of total cover. |
| Barren Land | Barren areas of rock/sand/clay, vegetation <15% of total cover. |
| Deciduous Forest | Areas dominated by trees generally greater than 5m tall, and greater than 20% of total vegetation cover. More than 75% of the tree species shed foliage simultaneously in response to seasonal change. |
| Evergreen Forest | Areas dominated by trees generally greater than 5m tall, and greater than 20% of total vegetation cover. More than 75% of the tree species maintain their leaves all year. |
| Mixed Forest | Areas dominated by trees generally greater than 5m tall, and greater than 20% of total vegetation cover. Neither deciduous nor evergreen species are greater than 75% of total tree cover. |
| Shrub/Scrub | Areas dominated by shrubs less than 5m tall with shrub canopy typically greater than 20% of total vegetation. |
| Grassland | Areas dominated by grammanoid or herbaceous vegetation, generally greater than 80% of total vegetation. |
| Woody Wetlands | Areas where forest or shrubland vegetation accounts for greater than 20% of vegetative cover and the soil or substrate is periodically saturated with or covered with water. |
| Emergent Herbaceous Wetlands | Areas where perennial herbaceous vegetation accounts for greater than 80% of vegetative cover and the soil or substrate is periodically saturated with or covered with water. |

Table S3. Seven trait categories used for functional trait analyses (Boersma et al. 2014, Schriever et al. 2015).

| Trait | Description | Trait values |
| --- | --- | --- |
| Body size | - | <9mm, 9-16mm, >16mm |
| Functional feeding group | Primary feeding mode | Collector-gatherer, engulfer-predator, filter-feeder, piercer - plants, piercer - predator, scraper/grazer, shredder |
| Locomotion | Dominant movement or habit | Burrow, climb, crawl, interstitial, sessile attached, swim, surface skate |
| Dispersal capacity | - | Aquatic active, aquatic passive, aerial active, aerial passive |
| Respiration mode | - | Gill, integument, plastron/spiracle/vesicle |
| Voltinism | Number of generations per year | <1 generation/yr, 1 generation/yr, >1 generation/yr |
| Diapause | Capacity to enter a state of dormancy in response to adverse environmental conditions | Known diapause, possible diapause (inferred from literature or closely related taxa), no known diapause |

Figure S1.

Figure S1. Taxonomic and trait ordinations of the prey community. Non-metric multidimensional scaling ordination of lakes by the taxonomic and trait composition of the prey community (excluding predatory taxa over 10mm). A) Taxonomic ordination (NMDS: k = 2, stress= 0.194), B) Trait ordination (NMDS: k = 2, stress = 0.115). Vectors represent significant correlations between environmental variables or species abundances and the ordination space (Pearson correlation: p < 0.05). The only influential species/traits (| r | > 0.5) were snails in the family Physidae, indicated in A. Each ordination was rotated so that its first axis is parallel to a vector of damselfly abundance (“Coenagrionidae”) to facilitate comparison among plots.
